# Distinct features of PsbS essential for mediating plant photoprotection

**DOI:** 10.1016/j.xplc.2024.101179

**Published:** 2024-10-28

**Authors:** Lili Chen, Melvin Rodriguez-Heredia, Guy T. Hanke, Alexander V. Ruban

**Affiliations:** School of Biological and Behavioural Sciences, Queen Mary University of London, London E1 4NS, UK

**Keywords:** PsbS, qE, dimer monomerization, 3_10_ helix, H3, phenylalanine, ESMFold

## Abstract

For optimum photosynthetic productivity, it is crucial for plants to swiftly transition between light-harvesting and photoprotective states as light conditions change in the field. The PsbS protein plays a pivotal role in this process by switching the light-harvesting antenna, light-harvesting complex II (LHCII), into the photoprotective state, energy-dependent chlorophyll fluorescence quenching (qE), to avoid photoinhibition in high-light environments. However, the molecular mechanism by which PsbS acts upon LHCII has remained unclear. In our study, we identified the specific amino acid domains that are essential for PsbS function. Using amino-acid point mutagenesis of PsbS *in vivo*, we found that the activation of photoprotection involves dynamic changes in the oligomeric state and conformation of PsbS, with two residues, E67 and E173, playing a key role in this process. Further, the replacement of hydrophobic phenylalanine residues in transmembrane helixes II (F83, F84, F87) and IV (F191, F193, F194) with tyrosine revealed that phenylalanine localized in helix IV can play a significant role in hydrophobic interactions of PsbS with LHCII. Removal of the 3_10_ helix (H3) amino acids I74, Y75, and E76 did not affect the amplitude but strongly delayed the recovery of qE in darkness. Moreover, an AI-assisted protein-folding evolutionary scale model approach (ESMFold) was adopted to intelligently manipulate protein functions *in silico and thus streamline and evaluate experimental point mutagenesis strategies*. This provides new insights into the molecular architecture of PsbS that are essential for regulating light harvesting in higher plants.

## Teaser

Plants must swiftly switch between light-harvesting and photoprotective states for optimal photosynthesis, with the PsbS protein playing a key role. This study reports the identification of critical amino acid residues that are essential for PsbS function, such as glutamates E67 and E173, phenylalanines in transmembrane helix IV, and the H3 motif comprising I74, Y75, and E76. These findings offer new insights into the molecular mechanisms of PsbS and highlight the potential of AI-assisted protein folding models to guide experimental mutagenesis.

## Introduction

Photosynthesis is a dynamically regulated process that transforms light energy into chemical currency. Within plants, light-harvesting complexes (LHCs) absorb light energy, facilitating charge separation in the reaction centers (Croce and van Amerongen, 2014). The LHCs that surround photosystem II (PSII) consist of the major antenna (trimeric LHCII) and the minor antenna (monomeric CP24, CP26, and CP29) (Croce and van Amerongen, 2014). As light intensity increases, LHCs accumulate more excitation energy than required for carbon fixation, posing a risk of damaging the photosynthetic machinery and leading to PSII photoinhibition (Ruban, 2016). To mitigate this photodamage, plants have evolved an adaptive mechanism known as nonphotochemical chlorophyll fluorescence quenching (NPQ), which safely dissipates excess absorbed energy as heat. NPQ is activated and relaxed within seconds to minutes (Ruban, 2016). Energy-dependent chlorophyll fluorescence quenching, referred to as qE, represents the primary component of NPQ, adapting to the rapid light fluctuations encountered in nature. qE plays a vital physiological role in enhancing plant fitness and productivity (Kulheim et al., 2002; Kromdijk et al., 2016).

Photosynthetic light harvesting and electron transport occur within the energetic membrane known as the thylakoid, driving the formation of a transthylakoid proton gradient (ΔpH). This gradient not only fuels ATP synthesis but also triggers qE in the LHCs under high-light conditions. In plants, qE is critically dependent on the presence of the PsbS protein (Li et al., 2000). In addition, the reversible de-epoxidation of the xanthophyll pigment violaxanthin to zeaxanthin modulates qE kinetics (Li et al., 2000; Ruban, 2016). Recent studies have demonstrated that enhancing the abundance of NPQ induction and relaxation components, including the violaxanthin epoxidase and zeaxanthin de-epoxidase enzymes, as well as PsbS, can improve crop yields (Kromdijk et al., 2016; De Souza et al., 2022). PsbS, a small (22 kDa) homolog of light-harvesting proteins, does not bind pigments (Fan et al., 2015). The *Arabidopsis thaliana* knockout mutant *npq4-1* lacks qE (Li et al., 2000), while overexpression of PsbS enhances the amplitude of qE and accelerates both its activation and relaxation (Li et al., 2002; Zia et al., 2011). Despite extensive research on PsbS and its utility in improving crop yields, the molecular mechanism underlying PsbS function remains to be clarified.

The mutation of two specific glutamate residues (E69 and E173) leads to complete deactivation of PsbS (Li et al., 2004). Given that PsbS undergoes a reversible dimer-to-monomer transition (DMT) driven by ΔpH (Bergantino et al., 2003), it has been proposed that protonation of these Glu residues induces DMT, thereby triggering qE (Pawlak et al., 2020). A recent molecular dynamics study suggested that the H3 motif of PsbS undergoes a conformational change from a turn/coil to a 3_10_ helix in response to ΔpH (Liguori et al., 2019). It was hypothesized that qE may be stably induced in LHCs through interaction with this 3_10_ helix on PsbS (Liguori et al., 2019; Krishnan-Schmieden et al., 2021). Moreover, PsbS plays a structural role in thylakoid membrane dynamics (Goral et al., 2012; Sacharz et al., 2017), which coincides with qE induction and relaxation. We speculated that the high abundance of hydrophobic amino acid side chains, particularly those of phenylalanine, in PsbS may contribute to this phenomenon. However, *in situ* evidence for any of these proposed mechanisms of PsbS function is lacking. To address these aspects *in planta*, we generated a series of PsbS point/domain mutants in *Arabidopsis.* We selected transformants with PsbS mutant proteins whose abundance matched that of native PsbS in wild-type (WT) plants. These transformants were used to investigate real-time transitions between PsbS oligomeric states and to attempt to identify distinct features of the protein that define qE induction, intensity, and kinetic properties *in vivo*.

## Results

### Mutation sites in PsbS

**Figure 1:**
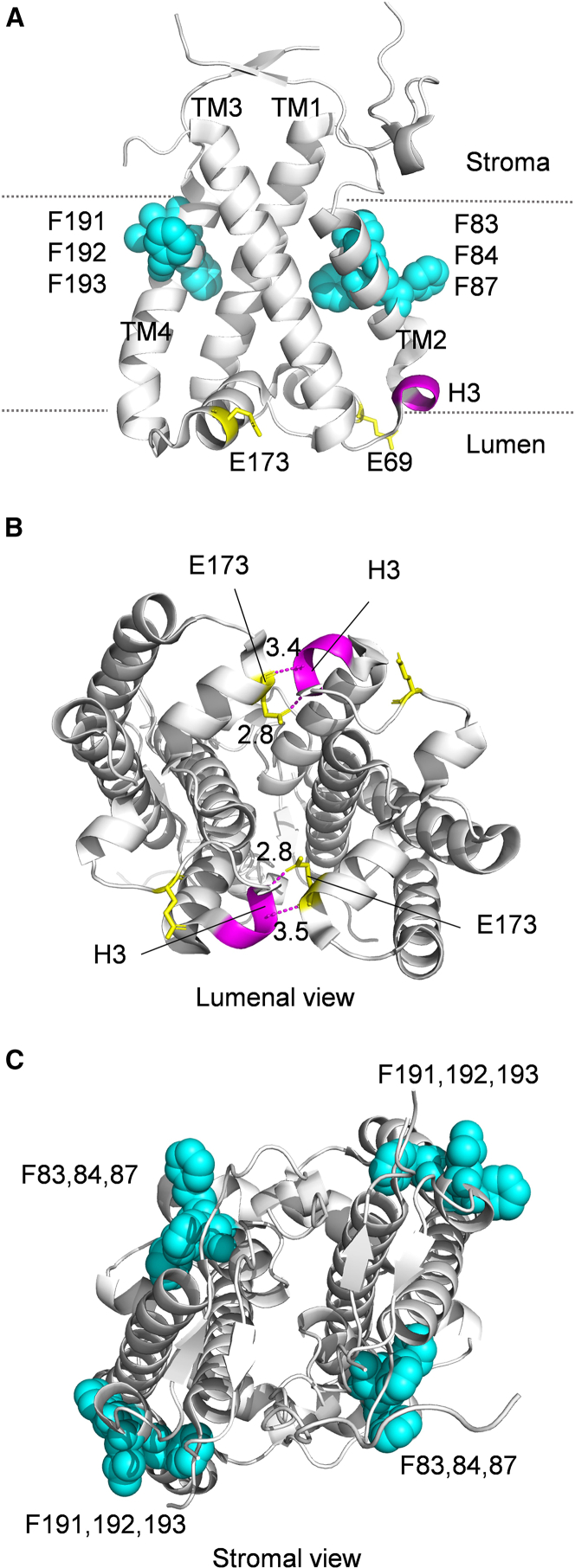
Localization of the mutation sites in the PsbS structure. **(A)** Monomeric structure of PsbS (PDB: 4R12) viewed from the membrane plane. The pH-sensing glutamates, E69 and E173, are shown as yellow sticks. The H3 motif is colored in magenta. The clustered phenylalanines in TM2 and TM4 are highlighted by blue spheres. **(B)** Lumenal view of the PsbS dimer, shown with the hydrogen bonds formed between E173 and H3 motifs in each of the monomeric PsbS proteins. **(C)** Stromal view of the PsbS dimer, with the localization of phenylalanine indicated.

Figure 1A shows the location of residues mutated and deleted in this study, mapped onto the crystal structure of spinach PsbS, which was resolved as a homodimer (Fan et al., 2015). The mutated domains are located either at the lumenal side or in the transmembrane region of PsbS (Figure 1A; Supplemental Figure 1). The glutamate residues E69 and E173 are in the middle of each of the two lumenal loops (Figure 1A) and were mutated into glutamines in the *E69QE173Q* mutant. The small H3 motif is located at the end of transmembrane helix 2 (TM2) facing the lumen, and it is composed of three amino acids, I74, Y75, and E76 (Figure 1A). Importantly, the structure indicates that four hydrogen bonds are formed at the lumenal side of the PsbS homodimer between E173 and the H3 motif (Figure 1B). The H3 motif was deleted to eliminate these lumenal interactions within the PsbS dimer.

To examine whether the transmembrane hydrophobicity of PsbS influences its activity *in situ,* multiple point mutations were performed in which phenylalanine was exchanged with the structurally similar, but polar, tyrosine residue (F to Y). Three clustered phenylalanine residues were selected for mutagenesis in TM2, F83, F84, and F87 (analogous to F83, F84, and F87 in the spinach structure shown in Figure 1A), and in TM4, F191, F193, and F194 (analogous to the hydrophobic structure constituted by F191, F192, and F193 in the spinach structure in Figure 1A). We considered that mutation of a fourth residue in this cluster, F192, would present too great a risk to the protein’s structural integrity. Its side chain was modeled to fold back into the protein structure, whereas all of the others have side chains that protrude from the protein into the lipid phase and are located at the periphery of the PsbS dimer (Figure 1A and 1C). The F-to-Y *Arabidopsis* mutants created by targeting TM2, TM4, or both were named *F83YF84YF87Y*, *F191YF193YF194Y*, and *F83YF84YF87YF191YF193YF194Y*, respectively.

### Effects of PsbS mutations on qE

**Figure 2:**
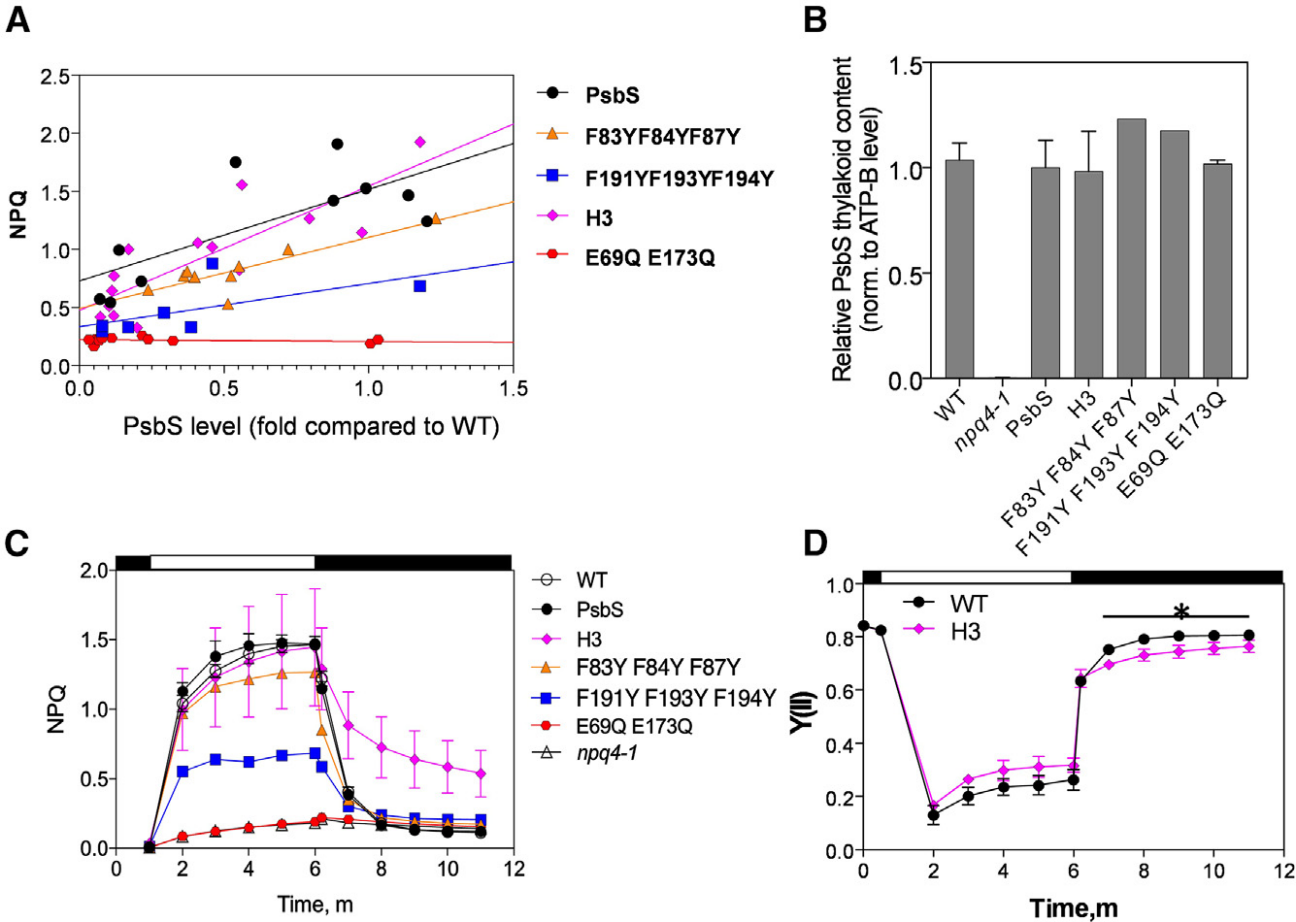
The amplitude and kinetics of NPQ in the PsbS mutants. **(A)** The correlation between NPQ amplitude and PsbS content. NPQ values were recorded after 5 min of illumination at 700 μmol photons m^−2^ s^−1^. For correlation analysis, experimental points were fit to the simple linear regression function (y = ax + b). **(B)** PsbS content in selected *npq4-1* rescue lines with mutant PsbS abundance closest to WT levels. **(C)** NPQ kinetics of the mutant PsbS lines indicated in **(B)**. White and black bars indicate the phases of illumination (700 μmol photons m^−2^ s^−1^) and darkness, respectively. Error bars in **(B)** and **(C)** indicate SD (*n* = 3 biologically independent samples for WT, *npq4-1*, PsbS, and H3; *n* = 2 for E69QE173Q; *n* = 1 for F83YF84YF87Y and F191YF193YF194Y). Fluorescence and biochemical analyses were performed in plants of the T1 generation with fully expanded leaves. PsbS levels were calculated from densitometry on western blots of thylakoid membrane proteins from the transformants. **(D)** Quantum efficiency of PSII in H3 mutants relative to the WT control. Measurements of Y(II) were performed on leaves at room temperature with 700 μmol photons m^−2^ s^−1^ of actinic light. Data are expressed as mean ± SD, *n* = 3 for WT control and biologically independent H3 lines with WT levels of PsbS. White and black bars indicate the phases of illumination and darkness, respectively. Asterisk indicates significant difference (*p* < 0.05).

Mutated *Arabidopsis PsbS* sequences were transformed into *npq4-1* under the control of the native PsbS promoter (∼1.8 kb). The WT *PsbS* gene was transformed in parallel to serve as the positive control. Mature leaves of T1 transgenic plants were analyzed for any correlation between NPQ amplitude and PsbS content (Figure 2A; Supplemental Figures 2 and 3). In *PsbS-*complemented and H3 lines, NPQ amplitude was proportional to the concentration of PsbS in the thylakoid membrane (Figure 2A). The *E69QE173Q* mutation completely deactivated PsbS, and NPQ was largely inhibited, independent of E69QE173Q-PsbS abundance (Figure 2A). Relative to protein abundance, NPQ in both the *F83YF84YF87Y* and *F191YF193YF194Y* lines was decreased (Figure 2A). The *F83YF84YF87YF191YF193YF194Y* mutant did not exhibit detectable PsbS accumulation in the membrane, which suggested a disruption of either protein folding or stability.

**Figure 3:**
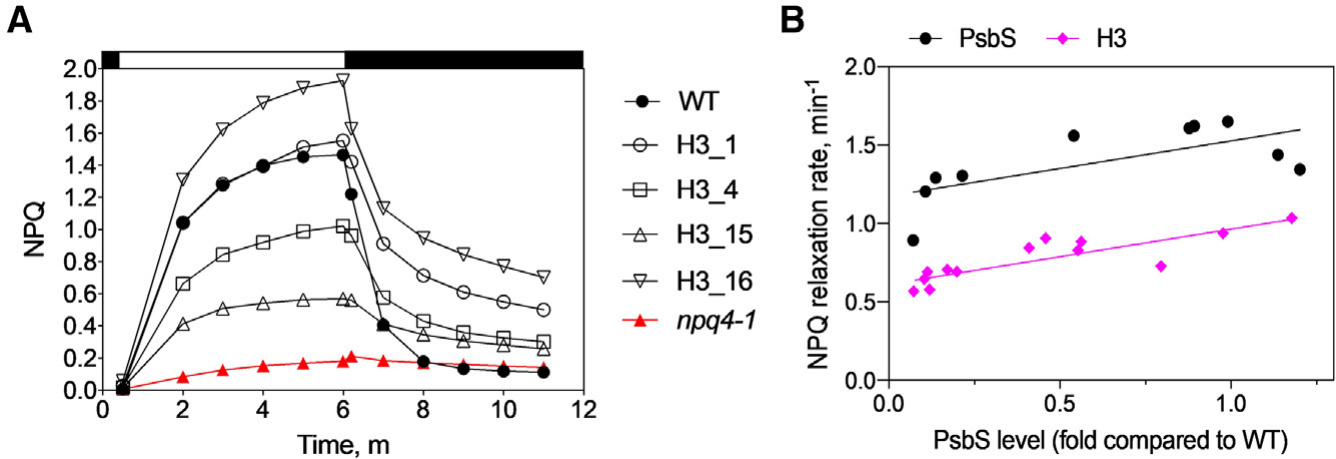
NPQ relaxation in the H3 mutants and PsbS-complemented lines. **(A)** NPQ kinetics of *H3* mutants of the T1 generation. NPQ was measured on leaves at room temperature with 700 μmol photons m^−2^ s^−1^ of actinic light. White and black bars indicate the phases of illumination and darkness, respectively. **(B)** The correlation between half time of NPQ relaxation rate and PsbS amount in the *H3* mutants and *PsbS*-complemented lines. The illumination cycle was the same as in **(A)**. The relaxation kinetics were fit to a one-phase exponential decay equation (Y = ae^−k^^X^ + b), where k is the rate of NPQ relaxation. For correlation analysis, experimental points were modeled with a simple linear regression function for *H3* (Y = 0.3516X + 0.6122) and *PsbS-*complemented lines (Y = 0.3517X + 1.174).

For each *npq4-1* transformation experiment, lines were selected in which mutant PsbS accumulated in the thylakoids to approximately the same abundance as native PsbS in the WT (Figure 2B), and the NPQ kinetics of these lines were analyzed (Figure 2C). The introduction of WT *PsbS* into *npq4-1* restored its qE kinetics to the WT level (Figure 2C; Supplemental Figure 4). *H3* lines showed the same NPQ induction kinetics as the WT but, interestingly, showed significantly slower NPQ relaxation (*P* < 0.05) (Figure 2C). qE was reduced by almost a third, and 5 min after the end of illumination, the remaining NPQ level was significantly higher (4.4-fold, *P* < 0.05) than that in the WT. The recovery rate of PSII quantum efficiency (Y(II)) was also significantly reduced (*P* < 0.05) (Figure 2D). In addition, the slower rate of NPQ recovery was independent of protein content, being observed in multiple *H3* lines with various levels of H3-PsbS protein abundance (correlating with NPQ amplitude) (Figure 3). In both *PsbS-*complemented and *H3* lines, the rate of NPQ relaxation was linearly and positively correlated with the amount of PsbS protein (Figure 3), but in all cases, the *H3* lines showed much slower rates of NPQ recovery (Figure 3). The *F83YF84YF87Y* plants showed a slight reduction in total NPQ amplitude relative to the WT by 27.3% (Figure 2C), whereas the *F191YF193YF194Y* plants showed a marked reduction in NPQ amplitude by 53.4% (Figure 2C). NPQ hardly developed in the *E69QE173Q* plants (Figure 2C), consistent with a previous report (Li et al., 2004).

### Transition of PsbS oligomeric states correlates with qE

**Figure 4:**
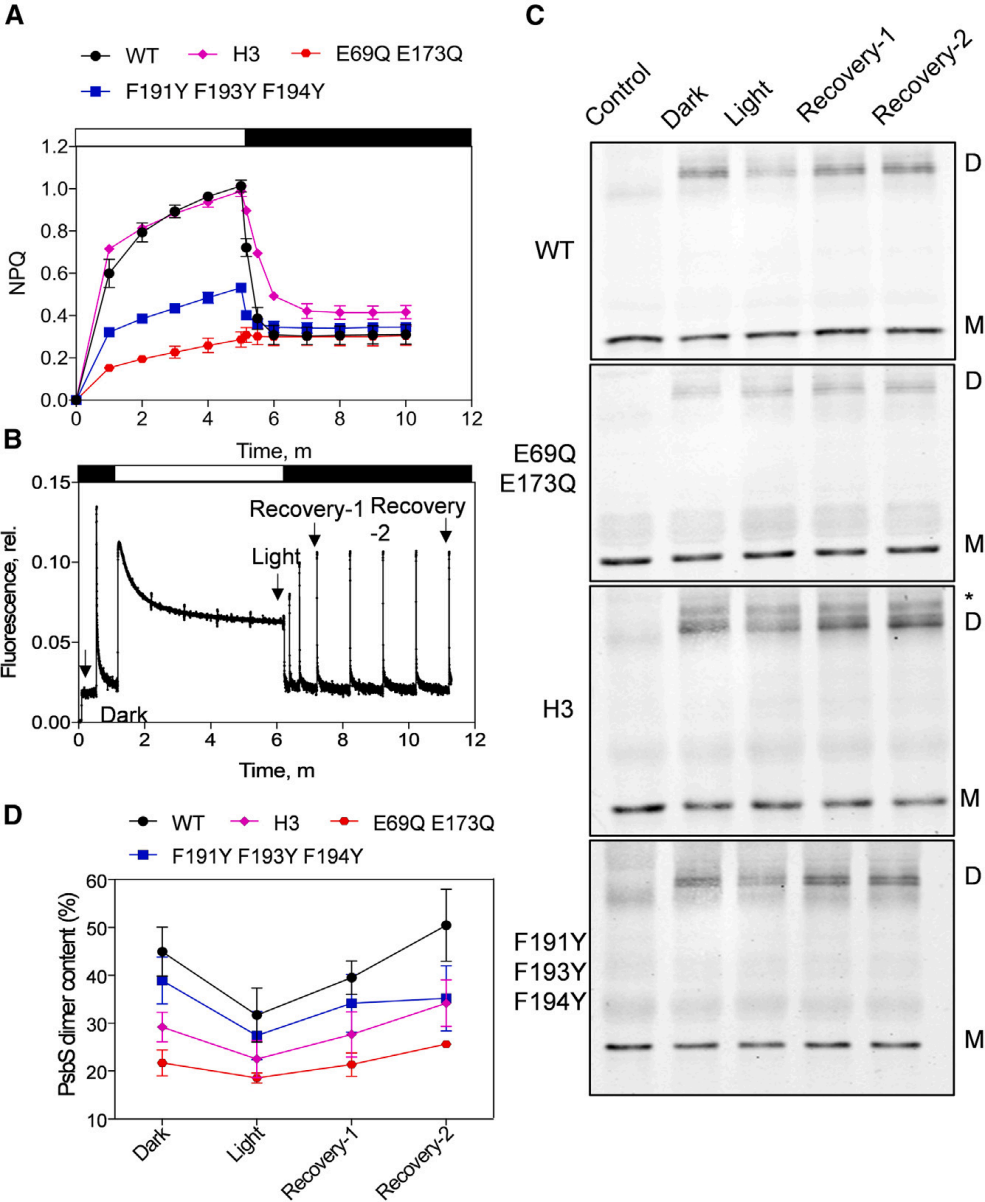
Oligomeric state transitions of PsbS during the kinetics of qE. **(A)** Quenching kinetics of thylakoids from PsbS mutants prior to cross-linking. **(B)** Representative PAM chlorophyll fluorescence trace of wild-type thylakoids, with the four states for cross-linking indicated. **(C)** PsbS oligomeric states in the thylakoids of mutants. Cross-linking of thylakoids was performed with 0.1 mM 3,3′-dithiobis (sulphosuccinimidylpropionate) (DTSSP), followed by SDS–PAGE and western blotting with PsbS antibody. D, PsbS dimer; M, PsbS monomer. Asterisk indicates the additional PsbS band. **(D)** Quantification of PsbS dimer contents in the four states of mutants relative to the WT control. For each fluorescence measurement and cross-linking, 60 μg Chl thylakoids were used. White and black bars in **(A)** and **(B)** indicate the phases of illumination (700 μmol photons m^−2^ s^−1^) and darkness, respectively. All values in **(A)** and **(D)** represent means of *n* = 4 biological replicates ± SD.

Because *E69QE173Q* has no qE and E173 is proposed to form hydrogen bonds between PsbS monomers upon dimer formation (Fan et al., 2015; Krishnan-Schmieden et al., 2021), it has been hypothesized that PsbS DMT is a key step for induction of photoprotection *in vivo* (Bergantino et al., 2003; Pawlak et al., 2020). To test this hypothesis, we investigated DMT activity by stabilizing PsbS dimers. This was achieved by chemically cross-linking PsbS with 3,3′-dithiobis (sulphosuccinimidylpropionate) (a hydrophilic 12 Å cross-linker), which effectively ligates the stroma-exposed lysine residues of PsbS monomers when they are in the dimer formation (Correa-Galvis et al., 2016). Optimal cross-linking conditions for probing *Arabidopsis* PsbS DMT during NPQ kinetics were established in isolated thylakoids of the WT (Supplemental Figure 5) and used in all further experiments. The thylakoids of mutant *PsbS* plants in the T2 generation were then extracted and their quenching kinetics evaluated with the same procedure (Figure 4A). Notably, the quenching phenotypes of the extracted thylakoids of all the mutants mimicked those in intact leaves (Figure 2). Four time points during NPQ kinetics were selected for cross-linking, corresponding to the dark-adapted, light, recovery-1, and recovery-2 states (Figure 4B). In this way, we aimed to capture the oligomeric states of PsbS on a timescale consistent with the quenching process. Cross-linked thylakoid samples were subjected to SDS–PAGE fractioning and western blot analysis. In the WT, DMT could be clearly observed, with a decrease in PsbS dimer band intensity in the light state (Figure 4C). After dark adaption, cross-linked PsbS dimer content relative to total PsbS was 44.9% ± 4.2% (Figure 4D); this decreased to 31.7% ± 4.6% in the light state and increased to 39.5% ± 2.8% and 50.4% ± 6.1% in the recovery states (Figure 4D). These results demonstrate that monomerized PsbS responds rapidly to the dissipation of ΔpH.

In the dark-adapted state, the *E69QE173Q* thylakoids showed significantly lower amounts of PsbS dimers (21.7% ± 2.4%) than the WT (*P* < 0.01) (Figure 4C). Dimer contents were also reduced to 18.6% ± 0.9%, 21.4% ± 2.1%, and 25.6% ± 0.8% of total PsbS in the light, recovery-1, and recovery-2 states, respectively (Figure 4D). Although some E69QE173Q-PsbS was retained in dimers, very little of this participated in DMT (77% less than WT, *P* < 0.01). By contrast, reversible DMT in the *F191YF193YF194Y* thylakoids was similar to that in the WT (Figure 4C), despite its greatly decreased NPQ phenotype, and dimer recovery was consistent with that of the WT. F191YF193YF194Y-PsbS did show variation from the WT in the period between the two recovery states, when recovery of PsbS dimers appeared saturated after 1 min (Figure 4D). For *H3* mutants, cross-linked PsbS was resolved into three bands, with an additional band above the PsbS dimer. When this was taken into account, dimer content in the dark state was reduced relative to that of the WT (*P* < 0.05). DMT was still detected, although densitometry indicated that it was 50.8% lower (*P* < 0.02) (Figure 4D). The total recovery to PsbS dimers was correspondingly reduced to 62.6% of the WT level (*P* < 0.05) (Figure 4D).

Recent advances in molecular dynamics studies and research involving recombinant PsbS proteins have enhanced our understanding of their functional mechanism. The mutant plants generated in this study provide an opportunity to investigate these proposed mechanisms *in vivo* and show profound deviations from the native PsbS in NPQ induction and relaxation activities. To determine whether misfolding of mutant PsbS sequences was responsible for these changes in NPQ, we used the AI ESMFold approach to recreate the tertiary protein structures of both WT and mutant PsbS proteins (Lin et al., 2023). ESMFold, a recent high-profile development in AI-based folding models, offers high-resolution, significantly accelerated folding predictions compared with the AlphaFold approach. ESMFold is applicable to the broadest range of protein structures. Because there is no PsbS crystal structure available for *Arabidopsis*, we compared the ESMFold-generated model to the known crystal structure of spinach PsbS. Supplemental Figure 6A represents an alignment of the PsbS crystal structure from spinach with the ESMFold-generated structure. They appear almost indistinguishable. We then aligned the mutant structures against the WT structure as depicted in Supplemental Figures 6 and 7. Upon initial inspection, these aligned structures appeared very similar, suggesting no significant alterations in the folding of the mutated protein structure. Supplemental Figure 8 presents the quantification of alignment quality, expressed as the root-mean-square deviation (RMSD) of atomic positions of the mutants from those of the WT, and plots the corresponding NPQ values against them. Surprisingly, the NPQ values are not negatively correlated with the RMSD; if anything, they exhibit a positive correlation. This suggests that alterations in the mutants’ overall structures were not significant enough to impair NPQ through misfolding and that the observed changes in this parameter were exclusively due to changes in the amino acid domains. Interestingly, the RMSD for the H3 mutant was greater than the RMSD predicted by the ESM accuracy (1.1 vs. 0.65). This difference is partly due to the altered tilt of the H2 helix (Supplemental Figure 6C). Nevertheless, the amplitude and kinetics of NPQ in the H3 mutant were similar to those in the WT. Thus, the mutation did not affect the induction or amplitude of quenching but significantly influenced the qE recovery kinetics. Consequently, combining *in vivo* point mutagenesis with AI-driven molecular modeling could enhance our understanding of the relationship between the atomic structure and the function of membrane proteins.

## Discussion

### Induction of qE

It has been reported that PsbS is activated by transition to a monomerized form, in which it is efficient in inducing LHCII quenching *in vitro* (Bergantino et al., 2003; Wilk et al., 2013; Pawlak et al., 2020). The work presented here shows that *E69QE173Q* plants are trapped in the qE-inactive state, as shown previously (Li et al., 2004) (Figure 2), but, in addition, we see that that the E69QE173Q-PsbS protein is in a significantly monomerized state compared with the WT (Figure 4). This is consistent with work on recombinant PsbS from *Physcomitrella patens*, in which mutation of the equivalent Glu residues also resulted in reduced dimer formation, particularly in the case of the single mutation equivalent to E173Q (Krishnan-Schmieden et al., 2021). In *E69QE173Q* plants, a small amount of dimer can still be seen (Figure 4), but the DMT of these remaining complexes is almost absent. It seems more likely that qE induction is associated with the DMT process but, most importantly, with the qE-active state of the PsbS monomer.

On the basis of molecular dynamics and two-dimensional infrared/nuclear magnetic resonance studies of recombinant PsbS, it has been proposed that PsbS dimers are stabilized by interactions between Glu173 and the H3 domain and that protonation of Glu triggers structural rearrangements in H3. This increases the ability of PsbS to interact with other proteins, such as LHCs, thereby inducing changes in the conformation of the PSII antenna (Liguori et al., 2019; Krishnan-Schmieden et al., 2021). Interestingly, removal of the H3 helix does not eliminate dimer formation, and DMT is retained, although at slightly lower levels than in the WT (Figure 4). Moreover, NPQ induction is indistinguishable between WT and *H3* plants (Figures 2 and 3). It may be that the H3 interaction with Glu173 can be substituted by other parts of the PsbS structure. The protonation of Glu69 and Glu173, followed by their repositioning into the membrane phase (Krishnan-Schmieden et al., 2021), therefore likely transduces changes in pH independent of H3 conformation.

In the WT, induction of NPQ is associated with the migration of PsbS from the grana core to the grana margin (Teardo et al., 2007; Correa-Galvis et al., 2016) and the dissociation of the PSII–LHCII core complex into LHCII trimers (Bergantino et al., 2003; Correa-Galvis et al., 2016). The *E69QE173Q* mutant of *Arabidopsis* reportedly lacks the LHCII-M-CP24-CP29 dissociation normally associated with qE (Betterle et al., 2009). It therefore seems likely that dimeric PsbS senses light-induced low pH in the thylakoid lumen through protonation of Glu69 and Glu173, promoting the PsbS lateral mobility associated with DMT and qE induction (Bergantino et al., 2003; Teardo et al., 2007; Correa-Galvis et al., 2016). We propose that the DMT process, rather than total PsbS monomer availability, is more likely to be a critical factor in this process.

### Amplitude of qE

Substitution of Tyr for Phe residues to reduce the hydrophobicity in TM2 and TM4 of PsbS reduces the amplitude of NPQ (Figure 2). DMT is retained in *F191YF193YF194YPsbS* thylakoids (Figure 4). These observations suggest that this hydrophobicity contributes to mechanisms that act independently and downstream of PsbS monomerization: namely, the functional mechanism. Monomerized PsbS gains the ability to interact with LHCII (Correa-Galvis et al., 2016; Sacharz et al., 2017), inducing its transition from the light-harvesting state to the quenching state (Croce and van Amerongen, 2014). This transition in LHCII could be achieved through either (i) direct docking via Phe residues, which would increase the pKa of glutamates and aspartates on LHCII and alter the lipid environment (Wilson et al., 2024), or (ii) Phe-mediated direct quenching of the chlorophyll excited state (Yan et al., 2008). The latter is less likely, as qE can still occur *in situ* without PsbS by enhanced ΔpH (Johnson and Ruban, 2011).

The Phe residues targeted in this study all protrude from the protein in the crystal structure (Fan et al., 2015), and their interactions must therefore be with extrinsic hydrophobic components, such as lipids or PSII antenna, rather than within the PsbS dimer. In the qE state, PsbS induces membrane reorganization (Johnson et al., 2011; Goral et al., 2012). In this state, PsbS interacts directly with LHCII (Correa-Galvis et al., 2016; Sacharz et al., 2017; Long et al., 2019), and LHCII trimers detach from the PSII core and form aggregates (Betterle et al., 2009; Holzwarth et al., 2009; Johnson et al., 2011). The reduced NPQ caused by Phe-to-Tyr exchange may therefore be due to altered PsbS–LHCII interactions, although no difference in protein interaction between WT and F191YF193YF194Y-PsbS could be detected using our protocol (Figure 4). Higher cross-linker concentrations were not used to probe this, as they resulted in high-molecular-weight aggregates and abolished DMT (Figure 4). Alternatively, PsbS has been reported to repel the lipid DGDG from LHCII, thereby increasing LHCII mobility and facilitating LHCII aggregation (Daskalakis et al., 2019; Wilson et al., 2024). The reduced NPQ in *F191YF193YF194Y* plants may therefore result from the reduced capacity of this less hydrophobic PsbS to bind DGDG.

It has been suggested that pH-responsive rearrangement of the H3 region in PsbS promotes inter-protein interactions with LHC proteins, leading to quenching (Liguori et al., 2019). These interactions are further enhanced during the induction of qE (Correa-Galvis et al., 2016; Sacharz et al., 2017). Interestingly, an additional band above the PsbS dimer was detected in the H3 thylakoids (Figure 4C), supporting the hypothesis that changes in the H3 region may facilitate the interactions of PsbS with other membrane proteins, most likely LHCs. However, these enhanced interactions did not affect NPQ amplitude (Figure 2).

### Relaxation of qE

It is believed that decreasing ΔpH, in combination with PsbS and epoxidation of zeaxanthin, controls the fast recovery of qE (Ruban and Horton, 1999; Zia et al., 2011; Long et al., 2019). The rapid relaxation of qE proceeds with similar kinetics in WT, *F191YF193YF194*, and *F83YF84YF87* plants but is disrupted by removal of the H3 domain from PsbS (Figure 2). This occurs despite *H3* plants having almost identical NPQ induction kinetics and amplitude to the WT. The rate of NPQ decay correlates with H3–PsbS content over a broad range of protein abundance *in vivo* (Figure 3), meaning that slowed recovery is almost certainly due to the action of the protein *per se* rather than to indirect action through xanthophyll pigments. To completely exclude variation in xanthophyll cycle activity and the amount of zeaxanthin accumulated during illumination, we analyzed leaf pigment composition following light treatment of the WT and H3 mutant line 1 (whose PsbS protein content is equivalent to that of the WT) using a high-performance liquid chromatography (HPLC) procedure (Ruban et al., 1999). Supplemental Figure 9 shows that there were no significant differences in xanthophyll pool size, de-epoxidation index, or synthesized zeaxanthin content between the H3 mutant and the WT. This result excludes altered xanthophyll cycle activity as a cause of slow relaxation kinetics in the H3 mutant. Therefore, it seems likely that the conformational changes detected in the H3 domain (Liguori et al., 2019; Krishnan-Schmieden et al., 2021) are specifically important for the reversibility of the quenching process. This specificity may result from a change in the tilt of the H2 helix (Supplemental Figure 6C). Such an alteration is a significant phenomenon and could be used in the manipulation of qE kinetics in plants, regardless of the mechanism of PsbS action. Further, PsbS has been shown to organize magnesium-induced attachment of LHCII to the PSII core (Kiss et al., 2008). Delayed NPQ recovery in *H3* plants may therefore be due to disrupted reattachment of PSII antenna to the core. One can speculate that the transition of H3 from a 3_10_ helix to a disordered loop upon increasing pH (Liguori et al., 2019; Krishnan-Schmieden et al., 2021) may thus regulate the dissociation of such PsbS–LHC interactions. This model is supported by the additional bands seen upon stabilization of H3-PsbS dimers (Figure 4). In the absence of H3, LHCII might therefore be sequestered away from PSII, impairing recovery.

**Figure 5:**
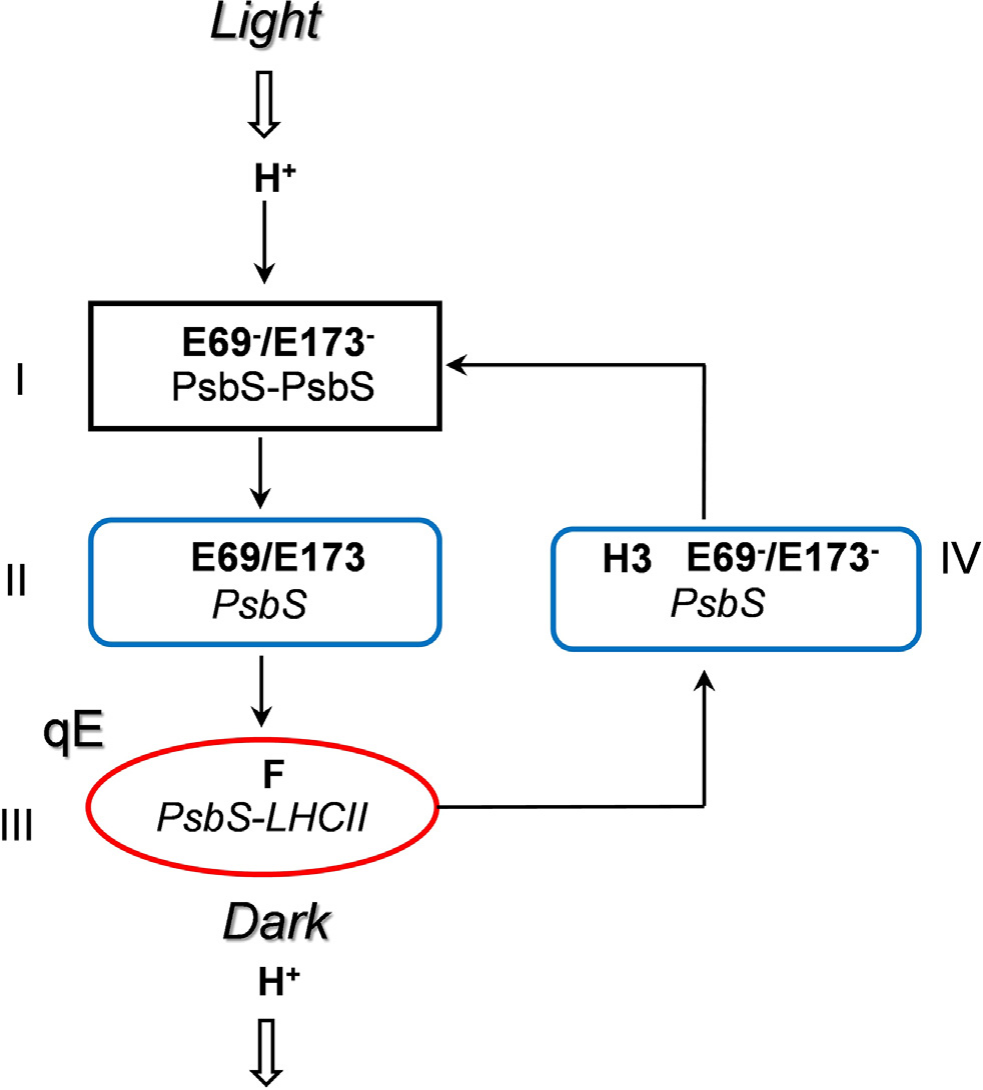
Model demonstrating a series of PsbS transitions for mediation of qE *in vivo*. Stage I: PsbS dimers sense low lumenal pH via glutamates E69 and E173 and rapidly monomerize. PsbS monomerization does not determine the rate of qE formation or amplitude, but it is essential for the activation of PsbS. Stage II: monomerized PsbS undergoes conformational change that may involve the H3 motif. Stage III: activated PsbS gains the ability to reversibly interact with LHCII, and its multiple hydrophobic F residues in TM2 and TM4 determine the amplitude of qE. Stage IV: the rate of inactivation of unprotonated PsbS and its removal from LHCII involves the H3 motif, by which PsbS controls qE relaxation. Unprotonated and unbound from LHCII, PsbS dimerizes. H^+^, protons in the thylakoid lumen; E69^−^/E173^−^, deprotonated glutamates; E69/E173, protonated glutamates; PsbS–PsbS, dimeric PsbS; *PsbS–LHCII*, active quenching complex; *PsbS* and PsbS, monomeric PsbS with/without conformational change.

In summary, we created and examined *Arabidopsis* plants in which the PsbS protein was replaced with specific mutated versions while maintaining WT abundance. We believe that the results offer new insights into the rapid photoprotective mechanism facilitated by this intriguing protein. Figure 5 illustrates a hypothesis for PsbS function based on our novel understanding of the four primary states through which the PsbS protein transitions during the induction and relaxation of NPQ. Acidification of the lumen, triggered by electron transport propelled by illumination, results in protonation of the E69 and E173 amino acids of dimeric PsbS (stage I). PsbS then undergoes monomerization and conformational changes (stage II). Apparently, the presence of PsbS monomers (Bergantino et al., 2003) is not sufficient for qE; a conformational change within the protein is required, as suggested by Krishnan-Schmieden et al. (2021). During stage II, PsbS interacts with LHCII, leading to activation of qE. This interaction could rely on specific phenylalanine residues, predominantly situated in helix IV. One of the most hydrophobic amino acids, phenylalanine forms a cluster of three residues that likely engage in robust hydrophobic interactions with LHCII. The transition to darkness or low-light conditions causes a decline in electron transport, consequently reducing ΔpH. Deprotonated PsbS then dissociates from LHCII in a process that involves the H3 helix domain. Notably, absence of this domain in the H3 mutant significantly delays the transition of LHCII into the light-harvesting mode. The experimental approach described here enables the generation of new hypotheses for testing in future studies and provides insights that may contribute to future work in crop improvement.

## Methods

### Plant material and growth conditions

Seeds of WT and *npq4-1* mutant *Arabidopsis thaliana* were vernalized for 3 days in the dark at 4°C after sterilization. The seeds were germinated in soil, and plants were grown in 10-h day/night cycles at 22°C with a light intensity of 200 μmol photons m^−2^ s^−1^. Plants used for *Agrobacterium*-mediated transformation were about 6 weeks old. The T_0_ generation of transformed plants was transferred to a short-day growth cycle (8-h day/night cycle) with a low light intensity of 100 μmol photons m^−2^ s^−1^. T1 and T2 generations of *Arabidopsis* transformants were planted and grown under 200 μmol photons m^−2^ s^−1^ with 10-h day/night cycles for 5 weeks before Chl (chlorophyll) fluorescence measurements and biochemical analyses.

### Site-directed mutagenesis and transformation

The WT PsbS gene was synthesized *in vitro*, coupled with the native 1.8 kb promoter sequence cloned from the genome of *Arabidopsis*. This WT PsbS DNA fragment, as well as the mutated fragments, were subcloned into the pSMAH vector for plant transformation. Codon changes in the PsbS gene were introduced *in vitro* using specific primers (Supplemental Table 1) and PCR. All mutations were confirmed by DNA sequencing (Supplemental Figure 10). The constructed vectors were transformed into *npq4-1* mutants by *Agrobacterium*-mediated T-DNA transformation (Clough and Bent, 1998).

### NPQ measurements

Chlorophyll fluorescence was assessed at room temperature using a DUAL-PAM-100 system (Walz Effeltrich, Germany). Both the attached rosette leaves and the isolated thylakoids were analyzed with the same actinic illumination of 700 μmol photons m^−2^ s^−1^. One illumination cycle was applied, consisting of 5 min of actinic light and 5 min of darkness. NPQ was calculated as (Fm – Fm′)/Fm′. NPQ measurements of thylakoids were performed in a quartz cuvette, with the samples gently stirred. Thylakoids (60 μg Chl) were resuspended in 2 ml of medium to reach a final concentration of 30 μg total chlorophyll ml^−1^. Total chlorophyll content was quantified from measurements of Chl *a* and Chl *b* based on their molar extinction coefficients (Porra et al., 1989).

### Thylakoid isolation

Thylakoid membranes for PsbS protein quantification were isolated as described previously (Casazza et al., 2001). For fluorescence measurements and chemical cross-linking, thylakoid membranes were isolated as follows. Leaves from dark-adapted *Arabidopsi*s were harvested and rapidly homogenized in a semi-frozen grinding medium (330 mM sorbitol, 5 mM MgCl_2_, 10 mM Na_4_P_2_O_7_, 2.5 mM EDTA, 40 mM D-iso-ascorbate [pH = 6.5]). Two or three short bursts of a Polytron homogenizer at ∼50% power were applied to avoid excessive homogenization. The homogenate was filtered through four layers of muslin and then through two layers of muslin with cotton wool into a beaker on ice. The filtrate was centrifuged at 4000 *g* for 3 min at 4°C. The supernatant was discarded, and the pellet was gently resuspended in four droplets of precooled resuspension medium (330 mM sorbitol, 5 mM MgCl_2_, 10 mM KCl, 50 mM HEPES, 2.5 mM EDTA [pH = 7.6]). A specific volume of break medium (5 mM MgCl_2_, 10 mM KCl, 50 mM HEPES, 2.5 mM EDTA [pH = 7.6]) was added to the suspension for 30 s, followed by addition of an equal volume of osmotic medium (660 mM sorbitol, 5 mM MgCl_2_, 10 mM KCl, 50 mM HEPES, 2.5 mM EDTA [pH = 7.6]). The suspension was centrifuged at 4000 *g* for 5 min at 4°C. After disposal of the supernatant, the pelleted thylakoid membranes were gently resuspended in 0.5 ml of resuspension medium.

### Chemical cross-linking

For chemical cross-linking, thylakoid membranes (equivalent to 60 μg Chl) were incubated for 1 min with 0.1 mM 3,3′-dithiobis (sulphosuccinimidylpropionate) in 2 ml of reaction buffer (450 mM sorbitol, 5 mM MgCl_2_, 10 mM NaHCO_3_, 10 mM EDTA, 20 mM HEPES, 20 mM sodium citrate, 20 mM MES, 100 μM methyl viologen [pH = 8.0]). The reaction was stopped by centrifugation at 14 000 rpm for 1.5 min, and the thylakoid membranes were resuspended in Laemmli buffer (Laemmli, 1970). The samples were further denatured at 37°C for 15 min (Schaller et al., 2011).

### SDS–PAGE and immunoblotting

Thylakoid membranes were mixed with Laemmli buffer (Laemmli, 1970) and then denatured at 37°C for 15 min (Schaller et al., 2011). After denaturation, a certain number of thylakoid samples were loaded into each lane of the gel together with a protein marker (10–250 kDa, P7719S, NEB). Hand-cast 12% acrylamide/bis-acrylamide gels were used for all SDS–PAGE analyses. Gel electrophoresis was performed at 4°C with a glycine buffer (25 mM Tris, 192 mM glycine, 0.1% SDS [pH = 8.3]). A constant voltage (100 V) was applied for the first 10 min to allow the proteins to enter the separating gel, and the voltage was increased to 180 V thereafter. Proteins in the SDS–polyacrylamide gel were transferred onto a nitrocellulose membrane by electrophoresis using a constant current (400 mA) for 1 h at 4°C. Immunoblotting was performed by incubation with primary antibodies specific for the *Arabidopsis* PsbS protein (Agrisera, Sweden) and ATP-B (Agrisera, Sweden). PsbS and ATP-B signals were detected after incubation with a secondary goat anti-rabbit antibody (IRDye 800CW, 1:20 000) and visualized with the Odyssey Imaging System. Quantitative densitometric analysis of PsbS and ATP-B protein signals was performed with Image Studio Lite software. Content of PsbS protein was normalized to that of ATP-B.

### Pigment analysis

Carotenoids were extracted from dark-adapted mature leaves using 100% acetone (HPLC grade), followed by centrifugation at 11 180 *g* and 4°C and filtration through a 13-mm diameter, 0.2-μm polytetrafluoroethylene syringe filter (Whatman). To assess zeaxanthin induction, pigments were also extracted from mature leaves after an illumination treatment with 500 μmol photon m^−2^ s^−1^ for 1 h. Pigments were separated by reverse-phase HPLC as detailed in Färber et al. (1997). HPLC analysis was performed on a BioLC HPLC system (Dionex) with a LiChrospher 100 RP-18 (5 μm) column (Merck). There were three biological replicates of each sample, each with three technical replicates. The amount of each carotenoid in the sample was calculated as a percentage of the total carotenoid content.

### Statistical analyses

Statistical significance was evaluated by two-tailed Student’s *t*-test. Fitting of NPQ relaxation data and correlation analysis were performed using Prism software.

## Funding

This work was supported by grants funded by a 10.13039/100010033Queen Mary University of London Ph.D. studentship to L.C. and a grant from The Leverhulme Trust to A.V.R.

## Acknowledgments

We thank Dr. Vasco Giovagnetti and Dr. Sam Wilson for assistance with SDS–PAGE, western blotting, and thylakoid isolation. We thank Dr. Vasco Giovagnetti for advice and fruitful discussions. No conflict of interest is declared.

## Author contributions

A.V.R. and G.T.H. conceptualized and L.C. designed the study; L.C., M.R.-H., and A.V.R. performed the experiments and analyzed the data; L.C. wrote the original draft; and G.T.H., M.R.-H., and A.V.R. reviewed and edited the manuscript.

## Supplemental information

Document S1. Supplemental Figures 1–10 and Supplemental Table 1

Document S2. Article plus supplemental information
