# Supplementary material for: Distinct features of PsbS essential for mediating plant photoprotection
Source: Plant Commun. 2024 Oct 28;6(1):101179. doi: 10.1016/j.xplc.2024.101179 (PMC11783875; doi:10.1016/j.xplc.2024.101179)
Supplement: Document S1. Supplemental Figures 1–10 and Supplemental Table 1 [file mmc1.pdf]

Plant Communications, Volume *6*

Supplemental information

Distinct features of PsbS essential for mediating plant photoprotection

Lili Chen, Melvin Rodriguez-Heredia, Guy T. Hanke, and Alexander V. Ruban

```
                    transit peptide
PsbS A.thaliana   MAQTMLLT-SGVTAGHFL--------RNKSPLAQP--KVHHLFLSGNSPVALPSRRQSFV
PsbS S.oleracea   MAQAMLLMMPGVSTTNTIDLKRNALLKLQIQKIKPKSSTSNLFFS-PLPSSSSSSSTVFK

PsbS A.thaliana   PLALFKPKTKAAPKKVEKPKSKVEDGIFGTSGGIGFTKANELFVGRVAMIGFAASLLGEA   57
PsbS S.oleracea   TLALFKSKA-KAPKKVEKPKLKVEDGLFGTSGGIGFTKENELFVGRVAMIGFAASLLGEG   56

                              E69    H3        F83 F84 F87
PsbS A.thaliana   LTGKGILAQLNLETGIPIYEAEPLLLFFILFTLLGAIGALGDRGKFVDDPPTGLEKAVIP   117
PsbS S.oleracea   ITGKGILSQLNLETGIPIYEAEPLLLFFILFTLLGAIGALGDRGRFVDEPTTGLEKAVIP   116

                                                                          E173
PsbS A.thaliana   PGKNVRSALGLKEQGPLFGFTKANELFVGRLAQLGIAFSLIGEIITGKGALAQLNIETGI   177
PsbS S.oleracea   PGKDVRSALGLKTKGPLFGFTKSNELFVGRLAQLGFAFSLIGEIITGKGALAQLNIETGV   176

                               F191 F193 F194
PsbS A.thaliana   PIQDIEPLVLLNVAFFFFAAINPGNGKFITDDGEES   213
PsbS S.oleracea   PINEIEPLVLLNVVFFFIAAINPGTGKFITDDEEED   212
```

Fig. S1. Sequence alignment of PsbS from *A. thaliana* and *S. oleracea* indicated with the mutation sites. The transit peptide of PsbS is indicated as previously reported (5, 7) and highlighted green. The targeted mutation sites are nominated based on their positions in the sequence of spinach PsbS to keep the consistency with other literature (5, 7, 13). The modified amino acids include lumen-exposed glutamates, E69 and E173 (red), the H3 motif (magenta), the phenylalanine in TM2, F83, F84, and F87 (orange) or in TM4, F191, F193, and F194 (blue).

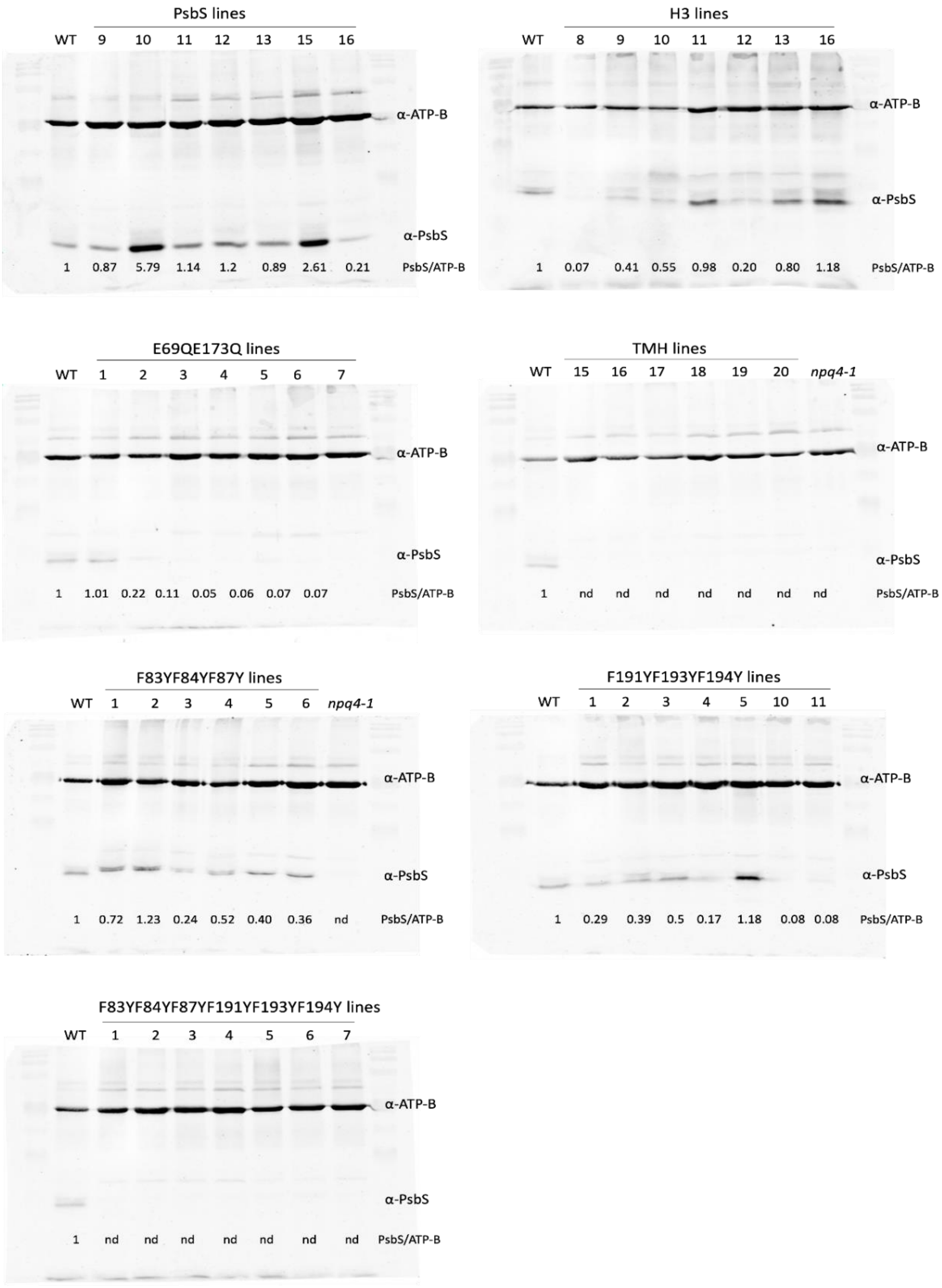

Fig. S2. The quantification of PsbS protein amount in the T1 generation of PsbS mutants. Equal amount of thylakoids (4 μg Chl) of WT and PsbS mutants were loaded into each lane for SDS-PAGE and western blot. The protein amounts of PsbS were determined by the densitometry values, and they were normalized to that of ATP-B. The numbers under each lane indicate the ratios of PsbS to ATP-B. TMH, the PsbS mutant with its 4th transmembrane helix deleted. nd, not detected.

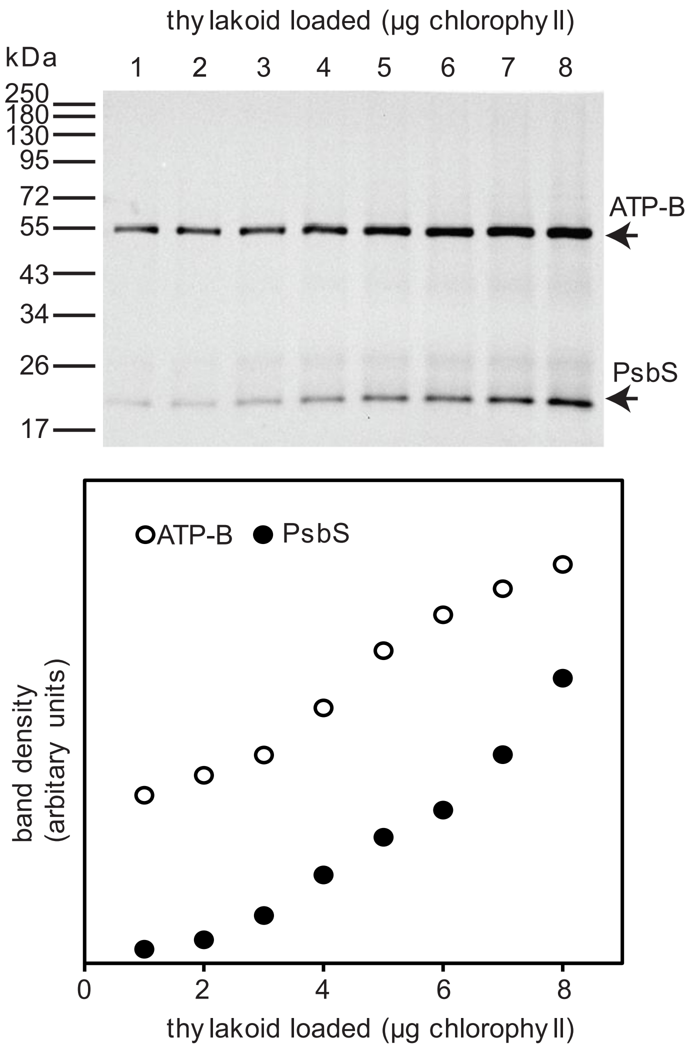

Fig. S3. Loading of SDS-PAGE gels for accurate detection of ATP-B and PsbS. Upper panel, the indicated quantity of isolated thylakoids, as measured by chlorophyll content, was loaded on SDS PAGE gels and blotted to nitrocellulose membrane before detecting with antisera raised against ATP-B and PsbS (indicated bands). Lower panel, densitometry was performed in ImageJ, and the area under the curve corresponding to the bands in each lane was plotted against chlorophyll content.

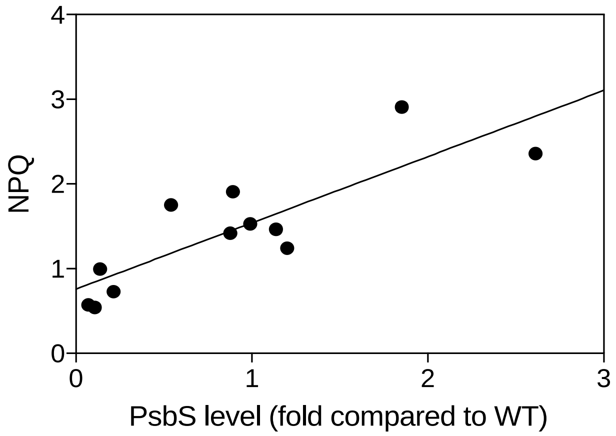

Fig. S4. Correlation between NPQ amplitude and PsbS content in *npq4-1* plants expressing the wild-type PsbS gene. Leaves of each T1 transgenic lines were illuminated for 5 min with 700 µmol photons m^{-2} s^{-1} of actinic light, and the maximal NPQ level was recorded. For each plant, the PsbS content was accurately estimated by SDS-PAGE and immunoblot analysis of isolated thylakoid membranes (4 µg Chl loaded). PsbS and ATP-B densitometry were detected by respective primary antibodies, and PsbS content was normalized to that of ATP-B. For correlation analysis, experimental points were fit into the simple linear regression function (y=ax+b).

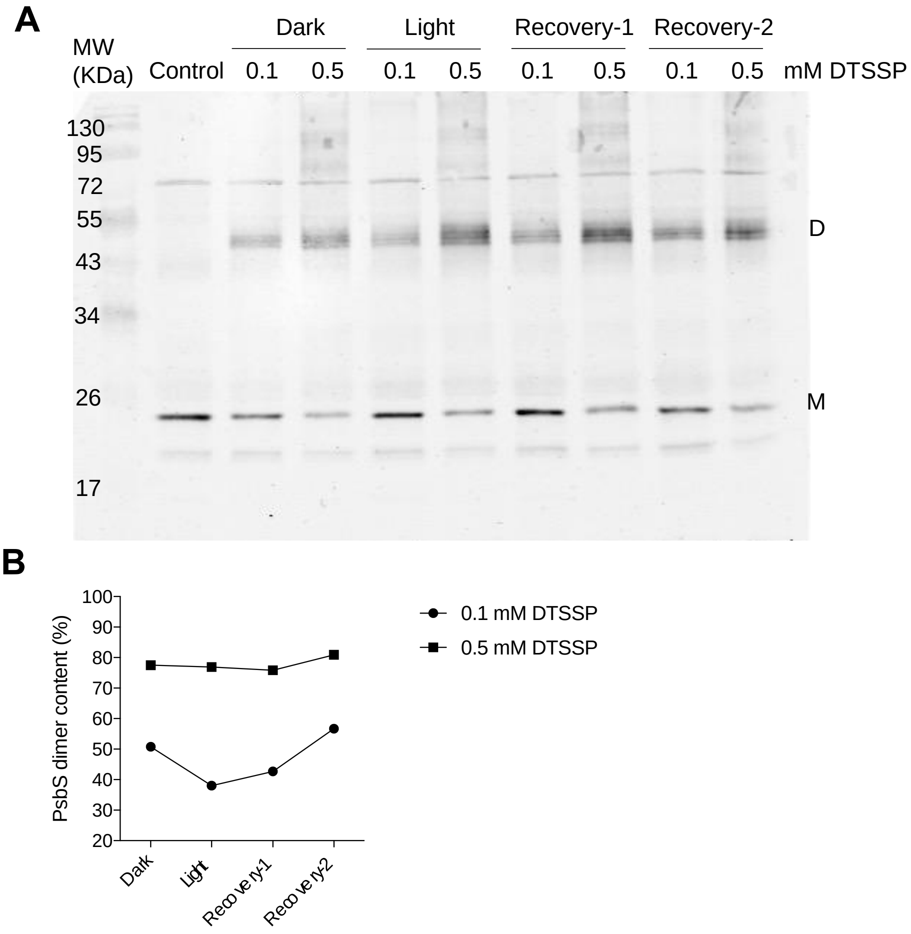

Fig. S5. Optimization of the crosslinking condition for WT thylakoids. (**A**) Crosslinking of WT thylakoids (60 μg Chl) performed in the dark, light, and recovery states with DTSSP (0.1 and 0.5 mM). The crosslinked thylakoid membranes were subject to SDS-PAGE and western blot analysis using PsbS antibody. (**B**) PsbS dimer contents in each of the four states under the two crosslinking conditions in (A). Actinic light illumination (700 μmol photons m^{-2} s^{-1}) was applied for 5 minutes, followed by relaxation in the dark for another 5 minutes. D, PsbS dimer, M, PsbS monomer.

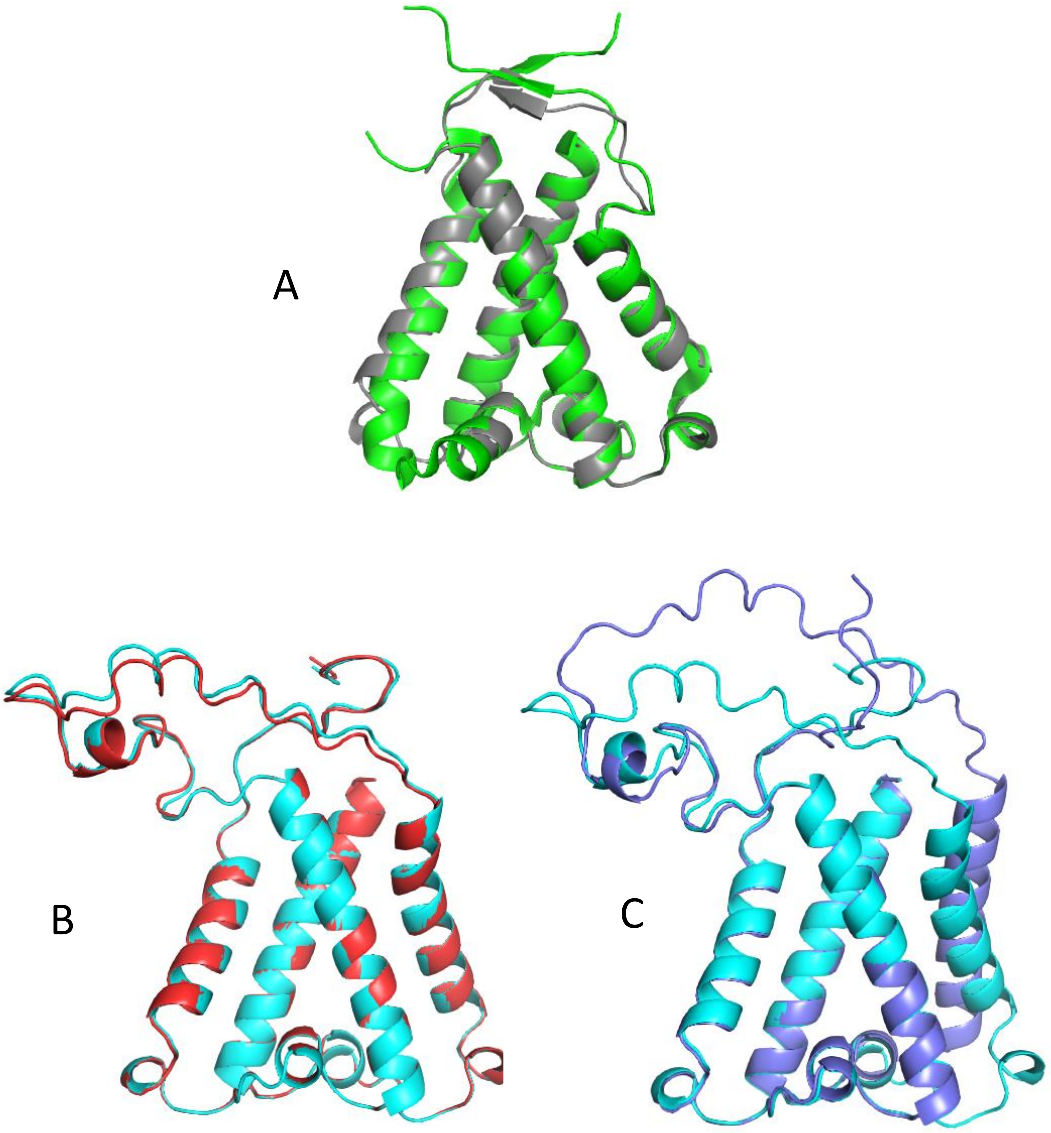

Fig. S6. PsbS structures generated from the primary sequences by AI ESM fold software. (A) PsbS from spinach crystal structure (green) vs PsbS folded by ESM fold (gray). (B) PsbS from Arabidopsis wild type (cyan) vs E69QE173Q mutant (red). (C) PsbS from Arabidopsis wt (cyan) vs H3 mutant (light blue).

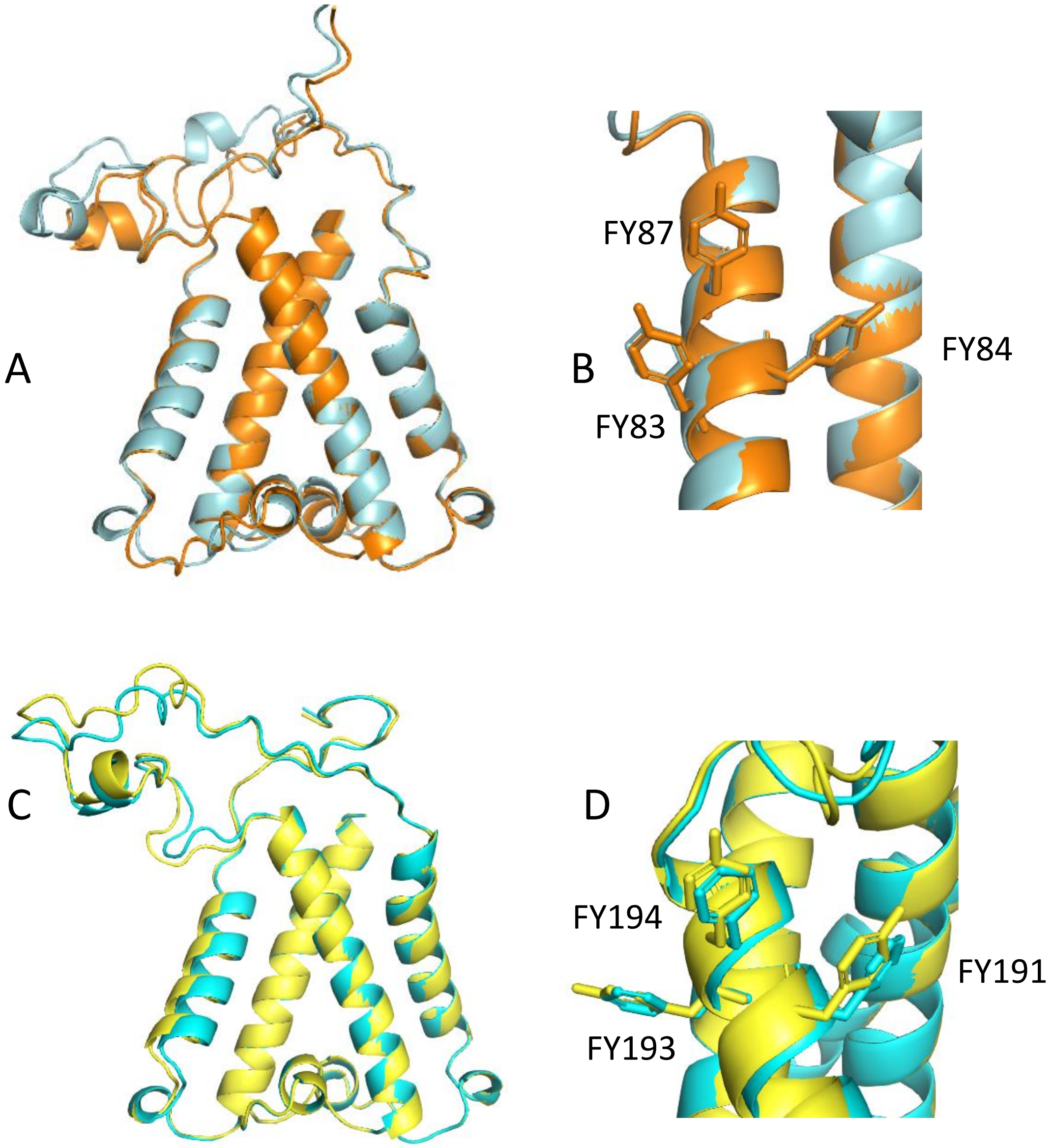

Fig. S7. PsbS structures from Arabidopsis generated from the primary sequences by AI ESM fold software. (A, B) PsbS wild type (cyan) vs F83YF84YF87Y mutant (orange). (C, D) PsbS wild type (cyan) vs F191YF193YF194Y mutant (yellow).

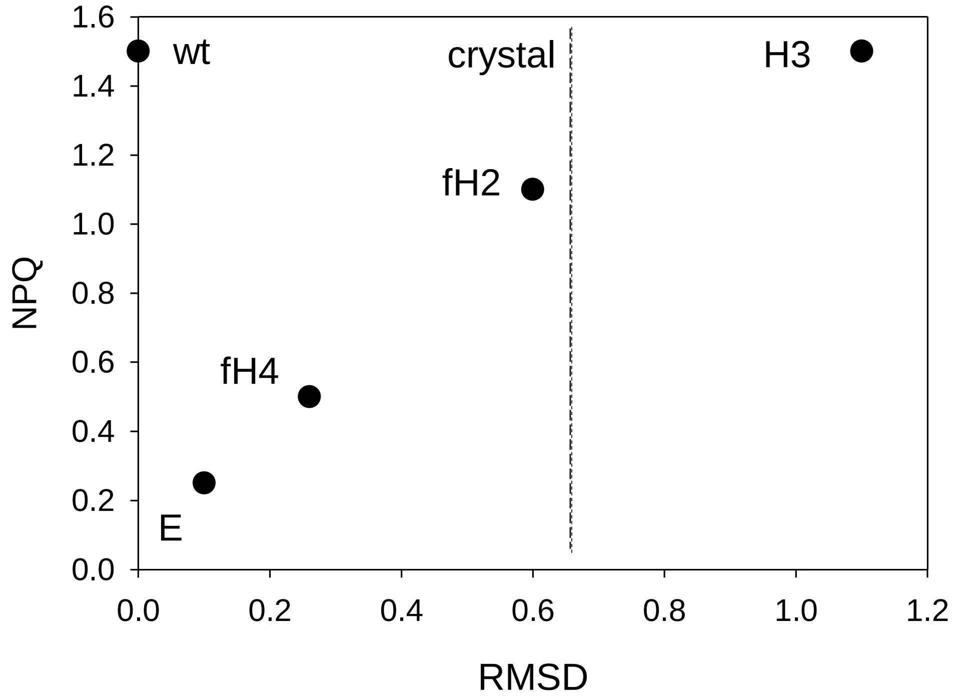

Fig. S8. Plot NPQ vs RMSD (random mean square deviation) from the wild-type structure for all PsbS protein mutant structures generated in this study by AI ESM fold software. *E, fH4, fH2, H3* stand for E69QE173Q, F191YF193YF194Y, F83YF84YF87Y and H3 loop mutants, respectively. *Wt* is a NPQ level for wild type Arabidopsis. *Crystal* and vertical dashed line indicates RMSD level comparing x-ray structure to ESM-generated structure of spinach PsbS.

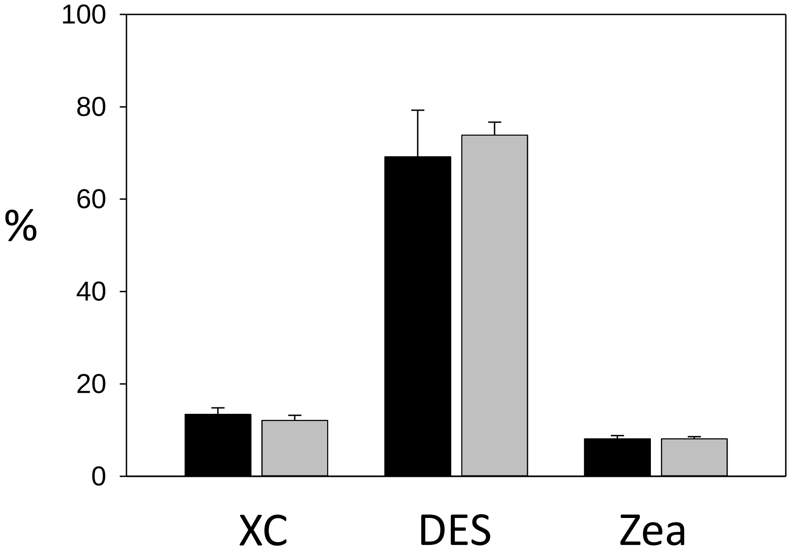

Fig S9. Xanthophyll cycle activity in the wild type (black bars) and H3 mutant (gray bars). XC is the percentage of the xanthophyll cycle carotenoids of the total carotenoid content of thylakoids. DES is the de-epoxidation index, calculated as $[(Z+0.5V)/(Z+A+V)]\cdot 100\%$, where Z, A, V are amounts of zeaxanthin, antheraxanthin and violaxanthin, respectively. Zea is percentage of zeaxanthin of the total carotenoid content. Data are expressed as mean ± s.d., n = 3. The difference between the wt and H3 mutant data was found to be not significant ($P>0.05$).

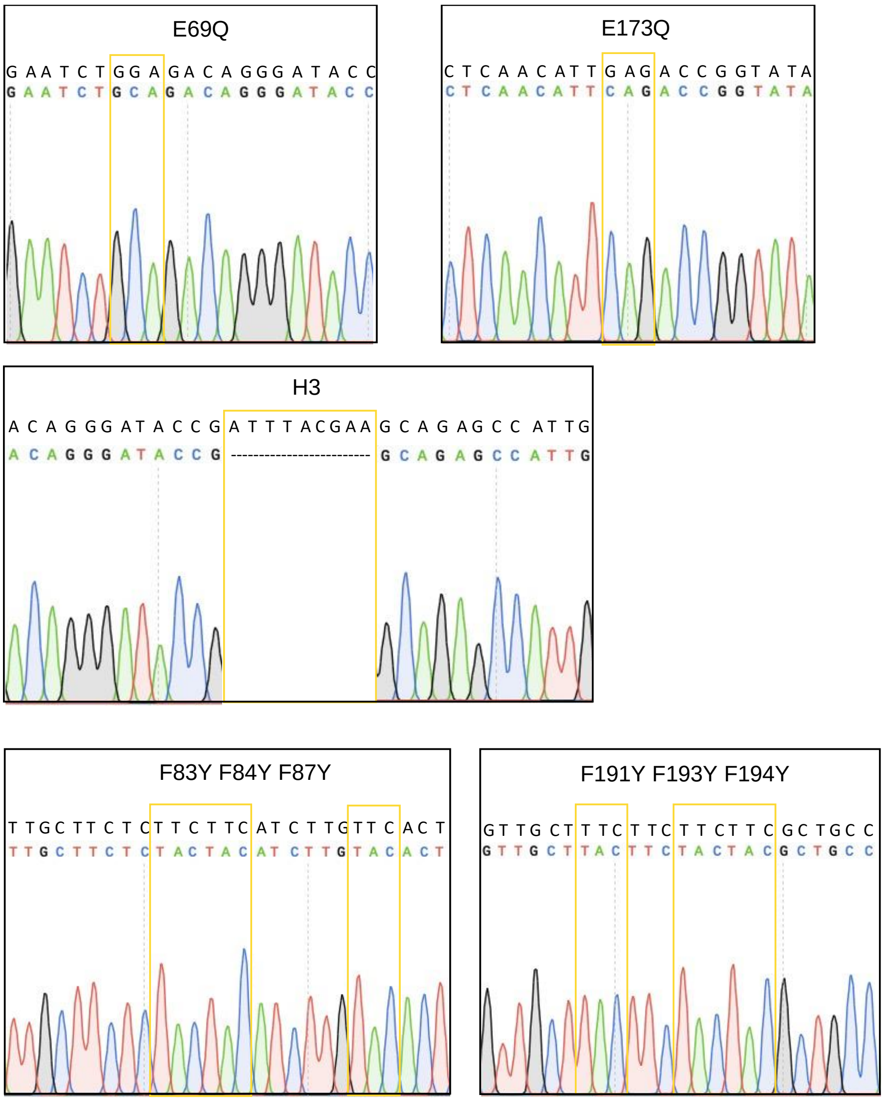

Fig. S10. Mutations verification by Sanger sequencing of PsbS constructs. In each panel, the upper and lower sequences represent the wild type PsbS DNA fragment and its mutated form, respectively.

Mutations	Forward primer	Reverse primer
E69Q	GAATCTGCAGACAGGGATACCGATTTAC	GTAAATCGGTATCCCTGTCTGCAGATTC
E173Q	ATTAGCTCAACTCAACATTCAGACCGGTATAC	GTATACCGGTCTGAATGTTGAGTTGAGCTAAT
H3	CCATTGCTTCTCTTCTTCATCT	CTCTGCCGGTATCCCTGTCTCC
F83YF84YF87Y	CCATTGCTTCTCTACTACATCTTGTACACTCTGTTGGG	CCCAACAGAGTGTACAAGATGTAGTAGAGAAGCAATGG
F191YF193YF194Y	CTTACTTCTACTACGCTGCCATTAATC	GATTAATGGCAGCGTAGTAGAAGTAAG

Table S1. Sequences of oligonucleotide primers used for site-directed mutagenesis of PsbS.
